# Red Cell Distribution Width in Rheumatoid arthritis

**DOI:** 10.31138/mjr.29.1.38

**Published:** 2018-03-19

**Authors:** Ziad S. Al-Rawi, Faiq I. Gorial, Anmar Abdulwahhab Al-Bayati

**Affiliations:** 1,2 Rheumatology Unit, Department of Medicine, Collage of Medicine, University of Baghdad, Iraq,; 3 Baghdad Teaching Hospital, Rheumatology Unit, Baghdad, Iraq

**Keywords:** Red cell distribution width, rheumatoid arthritis, inflammatory arthritis, autoimmune diseases

## Abstract

**Background:**

Rheumatoid arthritis (RA) is a chronic systemic autoimmune inflammatory disease, which affects the joints and other body systems. Red cell distribution width (RDW) is a part of the complete blood count (CBC) test and estimates erythrocytic variability.

**Objective:**

To evaluate RDW in RA and to assess the relationships of baseline demographic and clinical characteristics with RDW.

**Patients and Methods:**

A total of 111 patients with RA diagnosed according to the 2010 American College of Rheumatology/European League Against Rheumatism (2010 ACR/EULAR) classification criteria for Rheumatoid arthritis) and compared with 97 healthy individuals matched in age and gender as controls. Age, gender, body mass index (BMI), clinical diseases activity index (CDAI) and diseases activity score 28 using erythrocyte sedimentation rate (ESR) (DAS28-ESR), and diseases durations were recorded. Rheumatoid Factor (RF), anti-citrullinated peptide antibody (ACPA), complete blood count (CBC) and ESR were all measured.

**Results:**

The mean age for patients and controls were 46.53 ± 11.89 and 48.19 ± 12.06 years respectively (p=0.321). RDW was significantly higher in patients (14.5 ± 2.8%) compared with controls (12.4 ± 1.1 %) (p=>0.001). There was no significant correlation between various variables (age, Body Mass Index [BMI], disease duration, CDAI), DAS28-ESR, ESR, gender, RF, and ACPA) with RDW. The RDW had good ability to differentiate RA patients from controls (AUC=0.804, 95% CI=0.744 – 0.856. p=<0.001), while ESR had excellent ability to differentiate between RA patients and controls (AUC=0.926, 95% CI=0.882 – 0.958, p<0.001).

**Conclusions:**

RDW was significantly elevated in RA patients.

## INTRODUCTION

Rheumatoid arthritis (RA) is a chronic systemic autoimmune inflammatory disease characterized by inflammation of synovial joints in a symmetrical pattern leading to destruction of cartilage and bone, joint deformities, permanent functional impairment, and disability.^1,2^ Red cell distribution width (RDW), as part of a complete blood cell count, is a measured parameter performed by automatic hematology analyzer, and it is a laboratory parameter that estimates erythrocyte variability. Higher RDW values reflect greater heterogeneity in red cell sizes. In clinical and laboratory practice, RDW is widely used in combination with other hematology indices to identify the types of anemia.

Moreover, RDW levels as well as cytokine levels are associated with functional dependence in elderly people.^3,4^ Several linear evidences reported that increased RDW values have been associated with polymyositis, ankylosing spondylitis and multiple sclerosis, independently of infection, anemia and nutritional deficiencies.^5–7^

The novel index for inflammation, RDW, may be useful to estimate the disease activity in RA. In one study,^8^ RDW elevation was associated with RA disease, while another study^9^ reported that RDW could pose as a novel biomarker of inflammation in certain diseases due to its inexpensive and easy-to-access nature. However, standardization problems of the measurement assays are still important issues which need to be resolved. It could act as a strong biomarker of inflammatory conditions after standardization of measurement assays.

This study was designed to evaluate RDW in patients with RA and to assess their relationships with baseline demographic and clinical characteristics.

## PATIENTS AND METHODS

### Study design

This case-control study was conducted at the Rheumatology Unit of Baghdad Teaching Hospital in Medical City and Babil Centre for Biological Therapy in Merjan Teaching Hospital from August 2016 to January 2017.

### Sample selection

A total of 111 consecutive patients diagnosed to have RA according to the 2010 ACR/EULAR classification criteria for Rheumatoid arthritis^10^ were included in the study and compared with 97 healthy controls matched for age and gender. Informed consent was obtained from each participant included in this study according to the declaration of Helsinki. Ethical approval was obtained from the Ethics Committee in Medical Department, College of Medicine, Baghdad University.

Patients with the following conditions were excluded from the study: if they had other autoimmune connective tissue diseases like systemic sclerosis, systemic lupus erythematosus, and inflammatory arthritis like ankylosing spondylitis, inflammatory bowel diseases and psoriatic arthritis, acute or chronic infection, malignant diseases, renal and liver disease, haematological disorders such as iron deficiency anemia or had received blood transfusion during the past 4 months, cardiovascular and metabolic diseases, cerebrovascular disease, hypo- or hyperthyroidism, pregnancy or 6 months during postpartum period.

### Data collection and entry

Data entry of patients and controls were done using paper clinical research form (CRF) through interview and questionnaires. Age, gender, disease duration, and smoking status were reported. Height in centimeters and weight in kilograms were measured for all patients and controls, body mass index (BMI) was calculated according to the equation BMI=weight / height,^2^ disease activity and medications were recorded for all patients.

### Methods and data monitoring

Blood samples were taken from individuals in both groups to test for complete blood count (CBC), erythrocyte sedimentation rate (ESR), and red cell distribution width (RDW). Disease activity was measured using Clinical Disease Activity Index (CDAI)^11^ and Disease Activity Score 28 (DAS 28).^12^

### Statistical analysis

Anderson-Darling test was done to assess the normal distribution of continuous variables. Age, BMI, and diseases duration were normally distributed and presented as mean and standard deviation used. Categorical variables were presented as number and percentage. Independent t test (student t test) was used to compare the means of continuous variables between two groups. Chi square test used was used to compare the difference between the categorical variables of the patients and control groups. Binary logistic regression analysis was used to calculate the odd ratio (OR), when the independent variable can be categorized into 2 binary levels and compared to one dependent variable (in this case RDW). Linear regression analysis performed to assess the relationship between different variables and RDW, β (correlation coefficient or standardized beta is a representative of magnitude and direction of the relationship), β <0.25 weak, 0.25 – 0.5 mild, 0.5 – 0.75 moderate, >0.75 strong correlation. Negative sign indicates inverse relationship, but positive sign represents direct relationship.

Receiver operating curve was used to assess the validity of different parameters in separating cases with RA from controls and area under the curve (AUC), and its p value prescribed this validity (AUC ≥ 0.9 means excellent test, 0.8 – 0.89 means good test, 0.7 – 0.79 fair test; otherwise unacceptable). Trapezoidal method was used to calculate the curve. When a test is used either for the purpose of screening or to exclude a diagnostic possibility, a cut-off value with a high sensitivity may be selected; when a test is used to confirm a disease, a higher specificity may be required. SPSS 20.0.0, Minitab 17 and Graph Pad Prism 7.0 software package was used to make the statistical analysis. P value < 0.05 was considered statistically significant.

## RESULTS

A total of 208 individuals participated in the study. Of those, 111 were RA patients and 97 controls. The mean age of patients was 46.53 ± 11.89 years and that of controls was 48.19 ± 12.06. Females in RA patients were 98 (88.3%) and in controls were 76 (78.4%). There were no significant statistical differences between patients and controls in age and gender (p>0.05). Other demographic and clinical characteristics were shown in **Table 1**.

**Table 1.** Demographic and clinical characteristics of patients and controls.

**Variables**	**Control**	**RA**	**P value**
Number	97	111	
Age (years)	48.19 ± 12.06	46.53 ± 11.89	0.321
Gender			0.054
Female n (%)	76 (78.4%)	98 (88.3%)	
Male n (%)	21(21.6%)	13 (11.7%)	
BMI kg/m^2^	28.91 ± 4.04	29.34 ± 5.83	0.544
Smoking Hx positive n (%)			0.046
Current n (%)	26 (16.5%)	7 (6.3%)	
Former n (%)	5 (5.2%)	10 (9%)	
Never n (%)	76 (78.4%)	94 (84.7%)	
Disease duration (years)		8.67 ± 6.30	
Active disease n (%) using		107 (96.4%)	
DAS28		111 (100%)	
Active disease n (%) using		79 (71.2%)	
CDAI		67 (60.4%)	
RF positive n (%)		93 (83.8%)	
ACPA positive n (%)		111 (100%)	
Biologics n (%)		48 (43.2%)	
DMARDs n (%)		54 (48.6%)	
NSAIDs n (%)			
Steroids n (%)			

N: number, KG: kilogram, M^2^:sequare meter, DAS28:Disease Activity score28, CDAI: Clinical Disease Activity Index, RF :Rheumatoid Factor, ACPA: Anti Citrullinated Peptide Antibody, DMARDs: Disease Modifying Anti Rheumatic, NSAIDs: Non-Steroidal Anti Inflammatory Drugs, p value: Probability Value (<0.05) significant, %: percent.

**Figure 1.**
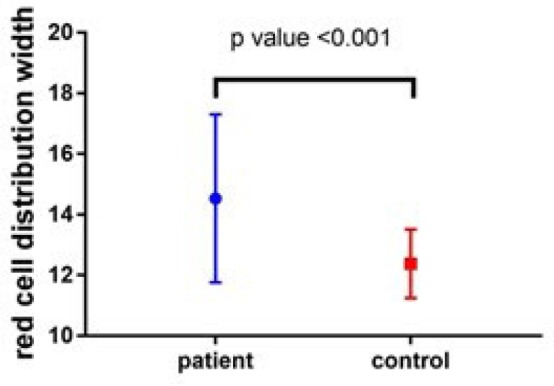
RDW for both patients and controls.

The RDW was significantly higher in RA patients compared with controls (14.5 ± 2.8 % vs 12.4 ± 1.1 %, p<0.001) as shown in **Figure 1**.

In RA patients, there was no statistical significant correlation between various variables (age, BMI, disease duration, DAS28, CDAI, ESR, gender, RF, and ACPA) with RDW (p>0.05) as shown in **Table 2**. The RDW had good ability to differentiate RA patients from controls while ESR had excellent ability to differentiate between RA patients and controls as illustrated in **Table 3**.

**Table 2.** Regression analysis to show the effects of demographic and clinical characteristic on RDW in RA patients

**Variables**	**Beta**	**OR**	**P value**
Age	−0.142		0.136
BMI	−0.072		0.450
Disease duration	−0.142		0.137
DAS28	−0.016		0.866
CDAI	0.093		0.971
ESR	0.076		0.427
Gender	−0.266	0.118	0.767
RF	−0.020	0.780	0.980
ACPA	−0.094	0.226	0.910

OR: odd ratio, beta: correlation coefficient (standardized), BMI: Body Mass Index, DAS28: Disease Activity Score 28, CDAI: Clinical Disease Activity Index, ESR: Erythrocyte Sedimentation Rate, RF: Rheumatoid Factor, ACPA: Anti Citrullinated Peptide Antibody, p value: Probability Value (<0.05). Age, BMI, disease duration, DAS28, CDAI and ESR linear regression were used.

Gender, RF and ACPA Both linear and binary logistic regression were used.

**Table 3.** ROC curve analysis of RDW and ESR as valid tests to differentiate patients from controls.

**Variables**	**AUC**	**95%CI AUC**	**P value**
RDW	0.804	0.744 – 0.856	<0.001
ESR	0.926	0.882 – 0.958	<0.001

AUC: Area Under Curve, CI: Coefficient Interval, RDW: Red Cell Distribution Width, ESR: Erythrocyte Sedimentation Rate, p value: Probability Value (<0.05), %: percent.

The optimal cut off point for RDW was >14.1%. At this cut-off point, this test had high specificity (95.88%) and low sensitivity (52.25%). Since its positive predictive value is 93.5%, this test is useful as a confirmation tool for diagnosis of RA in patients with clinical suspicion, while ESR at cut-off point of >26 mm/hr had high specificity (94.9%) and high positive predicative value (94.8%), indicating that as a test it had very good discrimination ability when used as a single test or in combination with prior assumptions. Also, ESR had high sensitivity compared to RDW and comparable specificity (95.88%, 94.85% respectively), as illustrated in **Table 4**.

**Table 4.** Validity of RDW and ESR to differentiate between RA and controls.

**Variables**	**Cut point**	**Sensitivity**	**Specificity**	**Accuracy**	**PPV**	**NPV**
RDW	>14.1 %	52.25%	95.88%	75.1%	93.5%	63.7%
ESR	>26 mm/hr	81.98%	94.85%	88.6%	94.8%	82.1%

PPV: positive predictive value, NPV: negative predictive value, RDW: Red Cell Distribution Width, ESR: Erythrocyte Sedimentation Rate, %: percent.

## DISCUSSION

This case control study showed that patients with RA have statistically significant higher RDW values compared with healthy controls. There was no significant effect of demographic and clinical characteristics on RDW. Both RDW and ESR were significant valid tests to differentiate between patients and controls with very high specificity and positive predictive value (PPV) more than sensitivity and negative predictive value (NPV) with good accuracy. This is clinically important in monitoring RA patients.

The high RDW in RA might be explained by the fact that RA is an autoimmune chronic disease which is often accompanied with anemia which may increase RDW.^13^ Another reason is that RDW may also be influenced by inflammation.^14^ Elevation in RDW has been reported in various inflammatory conditions in other studies.^15,16^

The data about the association between RDW and inflammation in literature are conflicting. Tecer et al^17^ reported that RDW were significantly higher in RA and RDW was similar to erythrocyte sedimentation rate and C-reactive protein to indicate inflammatory activity. In addition, RDW was correlated with DAS28. Yunchun et al^18^ observed an association between RDW and levels of inflammatory factors and autoantibodies in RA. They suggested that this association may be linked to the underlying proinflammatory state and increased oxidative stress, both of which correlate with impaired erythrocyte maturation. Also reported that RDW is a key player in the proinflammatory and proatherogenic status of RA, and may represent a useful marker to improve characterization of disease activity in RA patients. However, the correlation between RDW and DAS-28 was not observed in the study performed by Rodríguez-Carrio et al.^19^ This may be related to the small sample size and lower statistical power in that study.

A recent study performed by He et al^20^ investigated RDW as a potential laboratory parameter for monitoring inflammation in rheumatoid arthritis and showed that the level of RDW was elevated in RA patients and associated with inflammatory and antiinflammatory cytokines in RA patients compared to controls, and they suggested that the level of RDW could be a potential inflammatory marker for the monitoring of inflammation and disease progress in RA patients conveniently.

The major limitations of this study are absence of disease controls (e.g., SLE), the small sample, short duration and the fact that the patients were on treatment. Without disease controls, it is impossible to judge specificity of the observed shifts in RDW. However, this is the first study in Iraq that assessed RDW in RA with strict inclusion and exclusion criteria. Hemogram is a routinely used test for diagnosis and follow-up of rheumatic diseases. RDW is a part of hemogram and it does not incur any additional cost. It is a simple, cheap and widely available test which can be used on usual baseline and follow-up assessment of disease response to treatment.

In conclusion, RDW was significantly higher in RA patients compared with controls. This suggests that RDW may help in monitoring of RA and assessment of treatment response.
